# Insufficiency of Thyroid Hormone in Frog Metamorphosis and the Role of Glucocorticoids

**DOI:** 10.3389/fendo.2019.00287

**Published:** 2019-05-09

**Authors:** Laurent M. Sachs, Daniel R. Buchholz

**Affiliations:** ^1^Département Adaptation du Vivant, UMR 7221 CNRS, Muséum National d'histoire Naturelle, Paris, France; ^2^Department of Biological Sciences, University of Cincinnati, Cincinnati, OH, United States

**Keywords:** thyroid hormone, glucorticoids, metamorphosis, Amphibia Anura, crosstalk

## Abstract

Thyroid hormone (TH) is the most important hormone in frog metamorphosis, a developmental process which will not occur in the absence of TH but can be induced precociously by exogenous TH. However, such treatments including *in-vitro* TH treatments often do not replicate the events of natural metamorphosis in many organs, including lung, brain, blood, intestine, pancreas, tail, and skin. A potential explanation for the discrepancy between natural and TH-induced metamorphosis is the involvement of glucocorticoids (GCs). GCs are not able to advance development by themselves but can modulate the rate of developmental progress induced by TH via increased tissue sensitivity to TH. Global gene expression analyses and endocrine experiments suggest that GCs may also have direct actions required for completion of metamorphosis independent of their effects on TH signaling. Here, we provide a new review and analysis of the requirement and necessity of TH signaling in light of recent insights from gene knockout frogs. We also examine the independent and interactive roles GCs play in regulating morphological and molecular metamorphic events dependent upon TH.

## Introduction

Vertebrate life history transitions, such as birth or weaning in mammals, smoltification in fish, hatching in birds, and metamorphosis in amphibians, are associated with dramatic morphological and/or physiological changes underlain by striking maxima in several plasma hormone titers (1–6). Chief among the hormones involved are thyroid hormone (TH) and glucocorticoids (GCs), but other hormones with less extensive or recognized roles include prolactin, aldosterone, and insulin (7–11). Lack of GCs is not conducive to neonate survival in mammals (12), and lack of TH signaling precludes developmental progression in tadpoles (13), underscoring the critical importance of hormones during development. Typically, the actions of hormones during life history transitions are studied one hormone at a time, and when studying hormone interaction, the effect of one hormone's ability to affect the tissue sensitivity to other hormones is determined (14–16). However, other modes of hormone interaction are not well-characterized. Here, we focus on the extensively studied roles of TH and GCs in frog metamorphosis to gain insight into how hormones may interact to accomplish developmental changes.

The common thumbnail understanding of hormonal control of frog metamorphosis is that TH signaling is necessary and sufficient for metamorphosis and that GCs increase the rate of transformation via increasing tissue sensitivity to TH (13, 17–20). Similarly, signaling through TH receptors (TRs) is viewed as necessary and sufficient to initiate metamorphic events based on transgenic overexpression of mutant TRs (21–24). Current understanding of the molecular and developmental roles of TH and TR signaling has been summarized in the dual function model, where TRs act to repress TH-response gene expression in the absence of TH to prevent metamorphic events until TH becomes available in order to signal through TRs to induce TH response gene expression and accomplish metamorphic transformation (25–27). The current review will highlight previous and recent evidence suggesting modifications to this thumbnail sketch, namely that TH is required for complete tissue transformation in wild-type but not mutant animals lacking TRs, that TH signaling is not sufficient to accomplish frog metamorphosis, and that GCs do more than modulate TH tissue sensitivity.

## Analysis of the Requirement for TH/TR Signaling in Frog Metamorphosis

**Figure 1:**
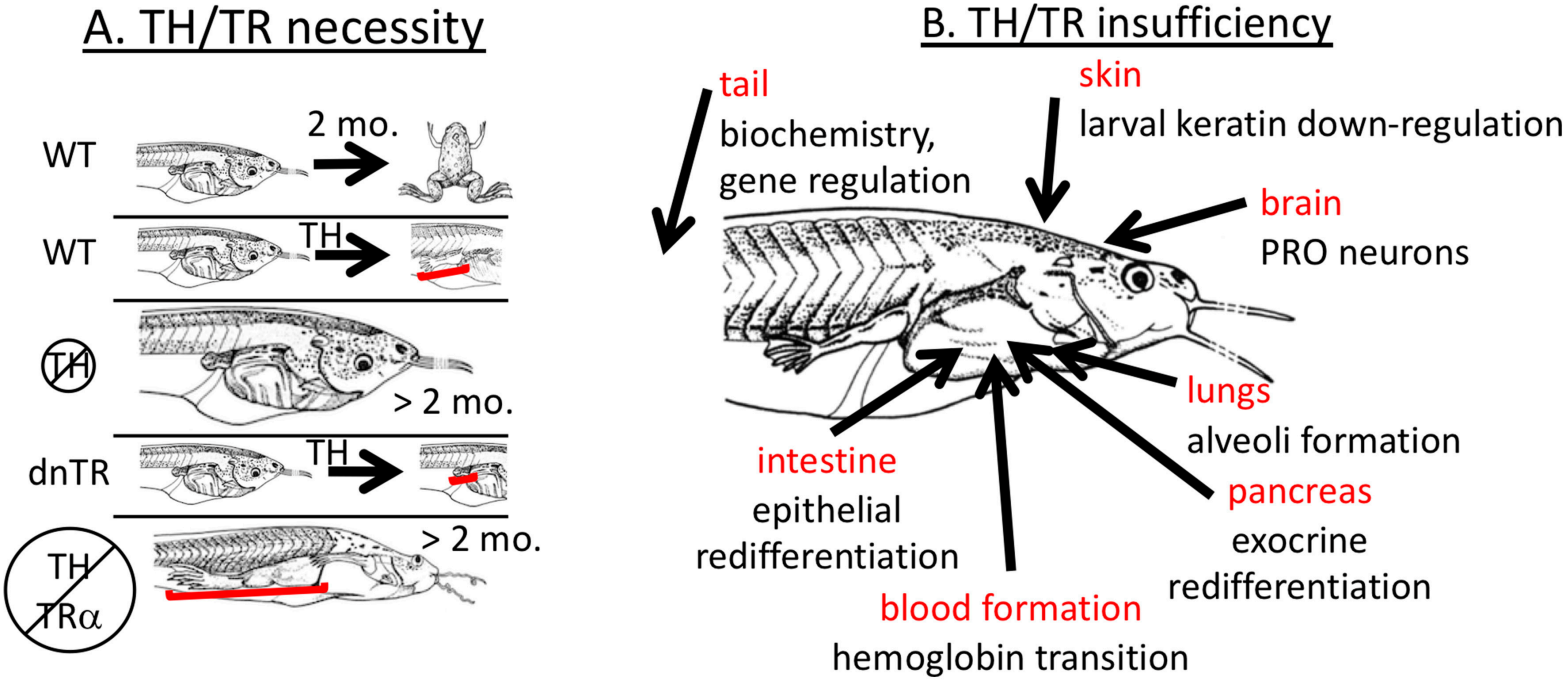
Necessity yet insufficiency of TH signaling in frog metamorphosis. **(A)** TH signaling is necessary. In wild-type (WT) tadpoles, metamorphosis is complete within 2–3 months, and tissue transformation, such as limb development, can be induced prematurely (3–5 days) by exogenous TH. Animals lacking TH are completely inhibited from metamorphic transformation but grow indefinitely in size. Tadpoles overexpressing dominant negative TH receptor (dnTR) do not exhibit limb elongation when treated with exogenous TH showing either that gene induction or at least lack of repression is required. When TH synthesis is blocked in TRα mutant animals (knockout), development of all tadpole tissues is stopped except limbs and skin which predominantly express TRα strongly in non-mutant animals indicating that lack of repression of TH response genes is necessary. Brackets highlight significant effects on limbs. **(B)** TH signaling is not sufficient. Many cases have been identified where exogenous TH is not sufficient to replicate natural metamorphosis. Each indicated tissue has metamorphic events that do not occur properly with just TH signaling. PRO = preoptic recess organ. See text for details.

Early experiments showed that TH is required for metamorphosis (13, 28). Removal of TH via embryonic thyroidectomy or treatment of tadpoles around or before the start of feeding with chemical inhibitors of TH biosynthesis (thiourea, propylthiouracil, potassium permanganate, methimazole) completely inhibits developmental progression beyond the foot paddle stage (Figure 1A). The inhibited tadpoles continue to grow at the same or faster rate than control tadpoles but external morphology, internal histology, and biochemistry remain larval with little if any indication of progress toward metamorphosis. Similarly, blockade of TR action by transgenic overexpression of a dominant negative TR inhibits metamorphosis when expressed all over the body and inhibits transformation of specific tissues when overexpressed in those tissues (Figure 1A) (21, 24, 29–34). These dominant negative TRs lack the last several C-terminal amino acids such that they cannot bind TH and thus maintain repression of TH response genes even in the presence of TH. Likely exceptions to a requirement for TH signaling to achieve the adult condition include lens crystallin transition, which appears to depend on tadpole size rather than stage (35) and gonad development where gonadal sex differentiation occurs on its own schedule irrespective of somatic developmental progression followed by oocyte and sperm production in the tadpole body in an extended absence of TH (36–40).

TH signaling is indeed required to accomplish metamorphosis, but how much signaling required is not defined. Two non-mutually exclusive models have been proposed to explain how much TH signaling is required, the “stoichiometric” model and the “threshold” model (41). A stoichiometric relationship between TH signaling and metamorphic progression implies that a certain sum total of TH signaling is required, which can be achieved by high levels of signaling for a short duration or lower levels over a longer duration (42). This model stems from the fact that the rate of induced metamorphic development is positively correlated with the concentration of exogenous TH. The stoichiometric model has been mistakenly contrasted with the threshold model of TH in metamorphosis where each developmental stage requires a certain minimum TH concentration in order to be achieved (43). This model stems from the fact that each tissue has its own threshold sensitivity to plasma TH level below which that tissue will not respond. Thus, near the threshold TH sensitivity for a tissue, the tissue transforms slowly with low levels of induced TH response genes, and at TH doses above the threshold, higher peak levels of TH response gene expression and developmental rates are achieved. In agreement with these models, spadefoot toad species with higher rates of metamorphosis have higher peak amounts of TH body content and higher levels of metamorphic gene expression compared to spadefoot species with longer larval periods (44, 45). Also, within a species, tadpoles reared in conditions that accelerate metamorphosis (e.g., low water) exhibit a higher peak in TH body content and TH response gene expression level (46).

Further insights into the role of TH signaling in metamorphosis came from *TR*α or *TR*β knockout animals. The result that removal of TH and the transgenic expression of dominant negative TRs block metamorphosis has been over-interpreted by virtually every expert in the field to mean that gene induction by TH is required for metamorphosis. Gene induction involves TH binding to TR and recruitment of co-activators that induce gene expression (20, 27). In the absence of TH, TRs recruit co-repressors to actively repress (i.e., “turn-off”) genes. In the absence of TRs, such active repression would not occur allowing “leaky” expression of TH response genes, but the level of such expression resulting from lack of repression is usually lower than that induced by TH binding to TR (47, 48). Thus, the blockade of metamorphosis due to lack of TH or overexpression of dominant negative TR has at least two possible interpretations. Either TH induction of genes is indeed required for metamorphosis, or alternatively lack of repression may allow enough expression of TH-regulated genes to enable metamorphosis. Rearing *TR*α knockout animals in methimazole resulted in full development of limbs and skin, suggesting that induction of TH response genes is not required for metamorphosis (Figure 1A) (48, 49). Thus, TH signaling is required in wild type animals, but results from *TR*α knockout animals suggest that the observed de-repression of TH response genes rather than TH-mediated induction of metamorphic genes may induce enough gene expression to allow metamorphic completion. It remains to be unequivocally demonstrated that limb and skin and any other organs can undergo full metamorphosis without induction of genes through TR by analyzing *TR*α/β double knockouts. The results with *TR*α knockout animals are consistent with the stoichiometric model, where a slight increase in TH response gene expression (de-repressed levels rather than induced levels) is enough to allow limb development to proceed to completion, albeit more slowly than normal. Similarly for *TR*β knockout animals, tail resorption is delayed, presumably because of the reduced TH signaling from loss of TRβ (50, 51).

The above results reveal consistent relationships between dose of TH, level of gene expression, and rate of developmental change, but the molecular mechanisms that determine how much signaling is required and for how long to achieve full tissue transformation are little understood. As a start, it has been estimated that two days of TH signaling is enough to achieve full TH signaling required for tissue destructive events but not constructive events (52), but this duration is likely dependent on TH dose, target tissue, and temperature. Removal of thyroidectomized tadpoles from TH treatments led to cessation of developmental progression after 2–3 days, where hind leg growth and tail regression came to a halt (53). More work is required to determine how the amount of TH signaling relates to the expression kinetics of TH response genes in the TH-induced gene regulation cascade that then controls the rate of metamorphosis.

## Analysis of the Sufficiency of TH/TR Signaling in Frog Metamorphosis

TH is considered to be sufficient for metamorphosis because addition of TH to premetamorphic tadpoles initiates virtually all known metamorphic changes (13). Continuous treatment of tadpoles with low doses or graded increases in TH dose over time enables animals to survive and complete metamorphosis precociously (53, 54). In addition, signaling through the TR appears to be sufficient to mediate the TH signal for metamorphic tissue transformations, because overexpression of a constitutively active mutant of TRα can initiate all the metamorphic events assessed (22). Addition of any other hormone by itself in the absence of TH, including GCs, aldosterone, and prolactin, has no known developmental effect during the larval period (7, 10, 11). Thus, it is commonly accepted that signaling through TH and TR is sufficient for all metamorphic transformations and that no other hormone is responsible or required. Despite this generalization, natural tissue remodeling is not always replicated by exogenous TH treatment. An obvious example is the jutting lower jaw and subsequent death typically within 7–10 days, prior to completion of metamorphic development in many organs when climax-level doses or higher of exogenous TH are given to young tadpoles (55). Additional discrepancies between natural and TH-induced metamorphosis have been observed in many organs, including lung, brain, blood, intestine, pancreas, tail, and skin (Figure 1B).

### Lungs

A striking example where TH treatment may not recapitulate the events of natural metamorphosis has been observed in lung transformation (56). In tadpoles, septa buds form and extend into the lumen of the sac-like lung forming numerous, thin-walled alveoli, a process that begins in premetamorphosis. The role of TH in lung development is not well characterized, but expression levels of *T*Rα and *TR*β increase in lung in TH-treated organ culture and reach a peak during natural metamorphosis in *Lithobates catesbeianus* (57). Similarly, exogenous TH induces the TH-response gene *Krüppel-like factor 9* (*klf9*) in organ culture in bullfrog, and reaches a peak at metamorphic climax in *Xenopus laevis*, though not in bullfrog (57, 58). In contrast to the natural septation process, treatment of premetamorphic bullfrog tadpoles with TH appeared to cause an abnormal thickening of the connective tissue in the lung wall and no septation (56). Addition work is required to examine what may explain this effect of TH and the potential role of GCs in lung morphological development.

### Brain

During metamorphosis, many TH-dependent changes occur in the central nervous system, including elaboration of the median eminence where hypothalamic axon terminals release hormones acting on the pituitary causing release of hormones that act to increase TH and GC levels (7, 59). In thyroidectomized or hypophysectomized (removal of pituitary) tadpoles, no monoamine-containing neurons appear in the preoptic recess organ of the hypothalamus, and neither catecholamine terminals nor capillaries appear in the median eminence (60, 61). Treatment with TH induced development of preoptic recess organ catecholamine neurons and capillaries in median eminence in the thyroidectomized bullfrog tadpoles, but surprisingly not in hypophysectomized bullfrog tadpoles even though external morphology was induced in both groups. Importantly, exogenous GCs, specifically corticosterone (CORT), induced the appearance of monoaminergic neurons in the preoptic recess organ in the hypophysectomized larvae but without causing morphological progress. Development of these neurons and capillaries appears to be the only GC-dependent and TH-independent metamorphic events known.

### Blood

During the climax of metamorphosis, larval erythrocytes containing larval hemoglobin are replaced by adult erythrocytes containing adult hemoglobin (62). Treatment of bullfrog tadpoles with TH induced minimal hemoglobin transition, and even after 2–4 weeks of treatment only partial adult hemoglobin synthesis and no decrease in larval hemoglobin occurred (63–65). Further, adult erythroblast proliferation was only minimally induced by TH, even though TH induced larval erythroblast apoptosis in the liver. However, erythroblast proliferation was substantially induced by TH plus CORT (though CORT by itself did not affect larval erythrocyte apoptosis) (66). Consistent with these results, inhibition of TH synthesis using propylthiouracil for over one year starting at early limb bud stage produced giant tadpoles of *Xenopus laevis* which exhibited a complete hemoglobin transition from larval to adult in the absence of morphological change (67). Similarly, the axolotl, a facultative neotenic species of *Ambystoma* salamanders, has adult rather than larval hemoglobin in a larval body (68) and the larval to adult hemoglobin transition occurs in thyroidectomized but not hypophysectomized larvae of the salamander *Hynobius* (69). Thus, even though production of adult hemoglobin can be induced by TH to a small extent, TH is not sufficient for the full larval to adult hemoglobin transition and is not necessary for the transition to occur.

### Intestine

During metamorphosis, the larval intestinal epithelium undergoes apoptosis, while adult epithelial cells from dedifferentiated larval epithelial cells proliferate, differentiate, and repopulate the intestinal epithelium to accommodate the change in diet from plant material to live prey (70). TH treatment of bullfrog tadpoles reared in thiourea decreased larval brush border hydrolytic enzyme activity, but adult levels of enzyme activity did not come about even after 15 days post treatment (71). TH treatment of small intestine cultured *in vitro* also caused larval cell degeneration (72), but adult epithelium failed to proliferate sufficiently (73), adult-type microvilli did not form (74), and adult-pattern lectin binding failed to occur (72). In addition, the adult epithelium achieved by natural metamorphosis and the epithelium achieved by TH treatment responded to GCs, specifically hydrocortisone, differently (75, 76). In particular, hydrocortisone increased intestinal digestive enzymes after natural metamorphosis but decreased them after TH-induction. However, *in-vitro* TH treatment of small intestine combined with the GC cortisol and/or insulin mimicked complete larval to adult epithelial transition reconstituting a brush border and exhibiting the supranuclear adult lectin binding pattern (72).

### Pancreas

During metamorphosis, the pancreas shrinks by 80% due to loss of zymogen granules and exocrine cell apoptosis (31, 77). Also, beta cells of the Islets of Langerhans exhibit a transient decrease in *insulin* mRNA expression though apparently without a decrease in beta cell number as they change from a larval to adult arrangement and cellular histology (32, 78). After climax, rebuilding the adult pancreas involves morphogenesis of the acini and ducts, redifferentiation of exocrine cells, and re-expression of endocrine hormones and begins around tail resorption when TH levels have already returned to baseline (31, 32). TH treatment mimics the morphological (reduction in pancreas mass) and biochemical (increased protein degradation and DNA synthesis) changes associated with remodeling of the larval pancreas that occur before metamorphic climax, but the increase in pancreas size and protein synthesis found in the natural remodeling process after metamorphic climax are not observed even after two weeks of TH treatment (though DNA synthesis does return) (79). Similarly, TH treatment induces the loss of larval alpha-amylase, but the normal replacement by adult alpha amylase does not occur (80). Partial pancreatectomy in premetamorphic tadpoles caused increased islet cell size and changed arrangement in ways reminiscent of metamorphic changes, leading to the view that islet remodeling may not be under TH control (81). However, islet remodeling appears to require TH-dependent remodeling of the exocrine pancreas, even when TH signaling is specifically blocked only in beta cells (32). These results suggest that pancreas resorption is stimulated by TH but that redifferentiation of newly proliferated exocrine cells accompanied by rearrangement of islet cells may not be dependent on TH.

### Tail

The sufficiency of TH in tail regression at the end of metamorphosis is not clear. Complete resorption of the tail is not observed upon prolonged treatment with moderate but effective doses of TH in premetamorphic tadpoles (41). However, treatment with a graded series of TH from low to high over successive days to mimic the developmental profile of endogenous plasma TH enables complete metamorphosis including tail resorption, and TH alone induces nearly complete tail shrinkage in culture devoid of other hormones (37, 53, 82). Even though GCs have no known action to induce tail regression, GCs synergize with TH *in vitro* to accelerate tail shrinkage (14, 83) and inhibition of GC signaling with amphenone B (a corticoid synthesis inhibitor) inhibited TH-induced tail resorption *in vivo* (84). In contrast, cortisol partly inhibited TH-induced reduction in DNA synthesis in tail epidermal cells (85), which is consistent with the observation that GCs by themselves increase tail growth *in vitro* (14, 83). In addition, CORT and TH have synergistic as well as antagonistic interactions at the level of gene expression (see below) (86). At the biochemical level, the role of taurine in tail regression is not known, but the amount of the atypical amino acid taurine in tail increases a few stages before tail regression during spontaneous metamorphosis but not in TH-induced metamorphosis (87). Also, beta-glucuronidase activity levels increase 3-fold more during spontaneous metamorphosis compared to TH-induced metamorphosis (88). On the other hand, cathepsin C increased in tail, gill, liver, lung, and kidney during natural metamorphosis, but TH treatment in premetamorphic bullfrog tadpoles induced cathepsin C activity only in the tail (89).

### Skin

Larval skin is glandless with three layers of uncornified epithelial cells containing cytokeratin 8 in the apical cell layer and larval keratin in the suprabasal skein and basal skein cell layers (90–92). Skein cells are strictly larval and have special intermediate filament bundles in them (called Figures of Eberth) (93). Metamorphosis results in the typical vertebrate cornified, stratified skin epithelium, which has a proliferative adult basal layer and expresses several adult keratins. The adult basal layer is derived from a series of three differentiation steps: first, basal skein cells change to adult keratin-positive basal skein cells which then change to larval (or pre-adult) basal cells associated with secondary connective tissue, and then these cells in turn change to adult basal cells (92, 94). In *in-vitro* skin culture, TH plus hydrocortisone but not TH alone reduced larval keratin synthesis in isolated primary epithelial cells after 4 days (95) but longer culture (9 days) with TH alone resulted in production of full adult skin except the secondary connective tissue did not form (96). Also, TH plus hydrocortisone-treated larval epidermal cells produced sheets of cornified cells as seen *in vivo*, while TH by itself induced only scattered single cornified cells (95). In addition, expression of adult keratin in basal skein cells can occur in culture in the absence of TH (96). Thus, the series of differentiation steps of the basal skin cells during skin transformation appear to involve a variety of TH- and GC-dependent and -independent steps.

## Modes of TH/GC Interaction

It is not known what explains the numerous cases where exogenous TH does not replicate events of natural metamorphosis. The cause of death after prolonged (7–10 days) TH treatment has been provisionally attributed to simultaneous initiation and highly abnormal rates of tissue transformations (97) or perhaps to thyro-toxicity because exogenous doses in the rearing water can achieve 5–6 times that amount within the tadpole body (98). Within a tissue, discrepancies between natural and induced metamorphosis may be due to incomplete organ competence to respond to TH, inappropriate TH dose, and/or requirement for other hormonal inputs. Future experiments are required to unequivocally establish any of these mechanisms. Here, we examine the possible requirement of GC signaling and interaction with TH for metamorphosis.

The best known role of GCs in metamorphosis is to synergize with TH to accelerate TH-induced metamorphosis in *Xenopus* (14, 17) and in axolotl (99). On the other hand, exogenous CORT by itself does not induce metamorphic development, and in fact inhibits growth and development in premetamorphic tadpoles (100, 101). Surprisingly, even when administered during prometamorphosis when endogenous TH is present, exogenous CORT still inhibits metamorphosis in *Xenopus laevis* (100) but accelerates development by itself in toad species (102, 103). Inhibitory effects of exogenous GCs administered during prometamorphosis may be acting at the level of the hypothalamus resulting in lower plasma TH, but this possibility has not been tested. Also, even though TH-response gene induction may not be required for metamorphosis (see above discussion), it is likely that CORT is required for survival through metamorphosis. In particular, only TH plus ACTH (adrenocorticotropic hormone, the pituitary hormone that stimulates production of GCs), but not TH alone, enabled survival through metamorphosis of hypophysectomized tadpoles (104). In contrast, lack of metamorphosis in hypothalectomized (hypothalamus removed, blocks stimulation of pituitary hormones required for TH and GC production) could not be rescued by treatment with TH and ACTH (13).

It is possible that the explanation for GC acceleration of TH-induced metamorphosis, death from lack of GC signaling, and the observed discrepancies between natural and induced metamorphosis may be related to TH tissue sensitivity. The increased tissue sensitivity induced by GCs (see below) can explain acceleration of TH-induced metamorphosis by GCs, where increased TH sensitivity would increase TH signaling and thus the rate of metamorphic development. Also, death from lack GC signaling and failure of exogenous TH to completely replicate natural development, which presumably includes a lack of a surge in GCs, may be due to lack of GC-induced increase in TH sensitivity, such that insufficient TH sensitivity may disallow development of a critical organ system before TH levels return to baseline or may lead to disruptions of the normal series of asynchronous developmental events (e.g., it would be problematic if leg development were not complete before tail resorption). On the other hand, high exogenous TH doses would presumably negate a need for GC-dependent increases in TH sensitivity. Thus, distinct from altered TH sensitivity, direct and required actions of GCs may explain death from lack of GC signaling and may also explain discrepancies between natural and induced metamorphosis. Examples of direct action of GCs different from just increasing TH sensitivity would be that GC-response genes may integrate with the TH gene regulation cascade and/or GCs may act on differentiation steps subsequent to differentiation steps accomplished by the TH gene regulation cascade. These modes of GC interaction with TH-dependent development, namely altered TH tissue sensitivity and direct effects of GCs, are discussed in the following sections.

## GC Regulation of TH Sensitivity

A well-researched mechanism to explain synergy between TH and GC signaling is the enhancement of tissue sensitivity to TH by GCs (14). Tissue sensitivity to TH is regulated by GCs through (1) altered deiodinase (TH metabolizing enzymes) expression and/or activity, (2) direct GC regulation of TH-response gene expression, such as the transcription factor *klf9*, and (3) indirect GC induction of TH receptor beta, which is one of the targets of Klf9. Reciprocally, the sensitivity to GCs can be enhanced in some tissues by TH via increased GC receptor expression.

### Effect on Deiodinases

Deiodinases are a family of enzymes that catalyze the release of iodine from TH leading to the production of T3 (the most active form of TH) from T4 (often considered a prohormone and binds with lower affinity to TH receptors) and to the degradation of T4 and T3 (105). Such TH activation by deiodinase type II (D2) and TH inactivation by deiodinase type III (D3) presumably allows fine control of intracellular hormone availability. Several studies have shown that the acceleration of TH-induced metamorphosis by GCs is partly due to the increased availability of TH in cells through GC effects on deiodinase expression or activity. First, GCs increased D2 activity in tadpole tissues associated with increased generation of T3 from T4 in *Lithobates catesbeianus* (16) and *Anaxyrus boreas* (106). Also, GCs decreased D3 activity in *Lithobates catesbeianus*, decreasing the degradation of T3 (16). Overall, these two actions of GCs contribute to the global increase in TH availability in metamorphosing tissues. Similarly in the neotenic amphibian, the axolotl (*Ambystoma mexicanum*), treatment with dexamethasone (a synthetic glucocorticoid) increased D2 activity and decreased D3 activity, and such changes were accompanied by an increase in plasma T3 levels (107). Using *Xenopus laevis* prometamorphic tadpoles, tail explant cultures, and frog tissue culture cells (XTC-2 and XL-15) (14) showed that *D2* mRNA levels were induced by GCs (and also by T3) supporting that the synergistic actions of TH and GC in metamorphosis occur at the level of expression of genes for D2, enhancing tissue sensitivity to TH (14). The mechanisms by which GCs regulate *D2* mRNA and enzyme activity levels are not yet defined.

### Effect on TRs

The level of *TR* gene expression is another central component of TH sensitivity (108). TH acts by binding to TR that functions as a ligand-activated transcription factor. The number of functional TRs expressed by a cell in large part determines the cell's sensitivity and responsivity to T3 (109, 110). TH itself can induce the expression of *TR* (autoregulation) in tadpoles, thus increasing the sensitivity to TH and driving the transformation process (111). In addition to autoregulation, other stimuli can influence the expression of *TR* (cross-regulation) (112). Such cross-regulation by GCs was first shown in bullfrog tadpole tail fins, where an increase in nuclear binding capacity for T3 was observed (113). A direct measure of *TR*β mRNA levels showed that CORT by itself upregulated *TR*β mRNA in the intestine, but not tail or brain, in *Xenopus laevis* (98), but *TR*β mRNA was synergistically upregulated by T3 plus GCs in tail explants, tail and brain *in vivo*, and tissue culture cells (14, 98). In contrast, T3 treatment or spontaneous metamorphosis lead to an increase in the number of T3 binding sites per nucleus in *Lithobates catesbeianus* red blood cells, but surprisingly this effect was abolished by dexamethasone (glucocorticoid receptor agonist) and sustained by dexamethasone plus RU-486 (glucocorticoid receptor antagonist) (114). Thus, the synergistic actions of TH and GC in metamorphosis involve increased *TR*β expression, thereby enhancing tissue sensitivity and responsivity to TH, though in a cell/tissue specific manner.

### Effect on klf9

TRs directly regulate numerous genes, some of which are transcription factors that in turn regulate the expression of other genes in a gene regulation cascade (20). One such transcription factor induced during metamorphosis is *Krüppel-like factor 9* (*klf9*) (115), which is a member of an evolutionarily conserved class of DNA-binding proteins that influence many aspects of development and physiology (116). Several members of this family were shown to be effectors of nuclear receptor signaling. Specifically, the KLFs can act as accessory factors for nuclear receptor actions, can regulate expression of nuclear receptor coding genes, and can be regulated directly by nuclear receptors. *Klf9* in particular is directly induced by GCs in a protein synthesis independent fashion exclusively via GR in tail, lungs, and brain (58, 117). It was further observed that GCs synergize with TH to superinduce the expression of *klf9* (112). This direct TH and GC regulation of *klf9* is evolutionary conserved as it also occurs in mammals (112). To explain how both TH and GCs synergize to increase *klf9* mRNA expression, a highly conserved 200 bp genomic region of the *Xenopus* and mouse *klf9* genes was identified 5–6 kb upstream of the transcription start site with binding sites for TR and GC receptor (GR) (112, 118). Characterization of this region has shown that TH increased the recruitment of liganded GR to chromatin at the enhancer element and that chromosomal looping allows the interaction of this far upstream enhancer region (5–6 kb) with the *klf9* promoter. This transcriptional mechanism of GC and TH interaction is known for just this gene, *klf9*, but there are likely other synergistic genes when considering that GCs synergize with TH to increase the rate of numerous morphological changes occurring during metamorphosis.

To reveal further how intertwined the relationship is between TH and GC interaction, Klf9 itself is involved in the autoinduction of TRβ by TH (119). Klf9 binds to GC-rich DNA regions present in the proximal promoter of the *Xenopus laevis TR*β gene to enhance its autoinduction by T3. Thus, Klf9 acts as an accessory transcription factor with TRs at TR direct target genes, which increases cellular responsivity to further TH action on developmental gene regulation programs (120). Furthermore, because *klf9* is also a direct GC target gene, GCs not only synergize with TH to induce *klf9* but also thereby increase tissue sensitivity to TH via Klf9 induction of *TR*β.

### Effects of TH on GC Signaling

TH affects GC signaling in at least two ways. First, T4-treatment, but not T3, increased whole body-GC levels in *Anaxyrus boreas* tadpoles, and a corticoid synthesis inhibitor prevented the stimulatory effect of T4 on GC production (106). Second, TH may regulate GR expression, at least in some tissues. During natural metamorphosis with its rising plasma TH titers, *GR* mRNA increases in the brain, lungs, and tail, but not intestine (58, 117). However, T3 treatment increased *GR* expression in the tail and decreased it in the brain (intestine and lungs were not assessed) (14, 98). Mineralocorticoid receptor (MR, the other nuclear receptor for GCs) increased during natural metamorphosis in brain, lungs, and tail and was shown to be inducible in the tail (58, 117). Thus, the synergy of T3 with GC during metamorphosis involves tissue-specific and T3-dependent regulation of *GR* transcripts.

## Diversity of GC and TH Crosstalk on Response Gene Expression

As shown above, exogenous TH does not always replicate natural metamorphosis. It is easy to add saturating amounts of TH to rule out insufficient TH signaling as the reason for the discrepancy in induced vs. natural metamorphosis. Also, the lack of metamorphic completion associated with lack of GC signaling (104) suggests that TH is not sufficient and that direct action of GCs not related to TH signaling is required. An important issue, then, is to distinguish between TH and GCs working simultaneously on the same cell vs. independent actions on cells of the same or different stages of differentiation. Also, at the level of gene expression, some changes in gene regulation in the presence of both TH and GCs are inconsistent with the synergistic morphological actions of the two hormones together. High throughput technologies provide a global perspective to help understand the mechanisms of interaction between TH and GCs.

### TH and GC Crosstalk: Gene Regulation Profiles

**Figure 2:**
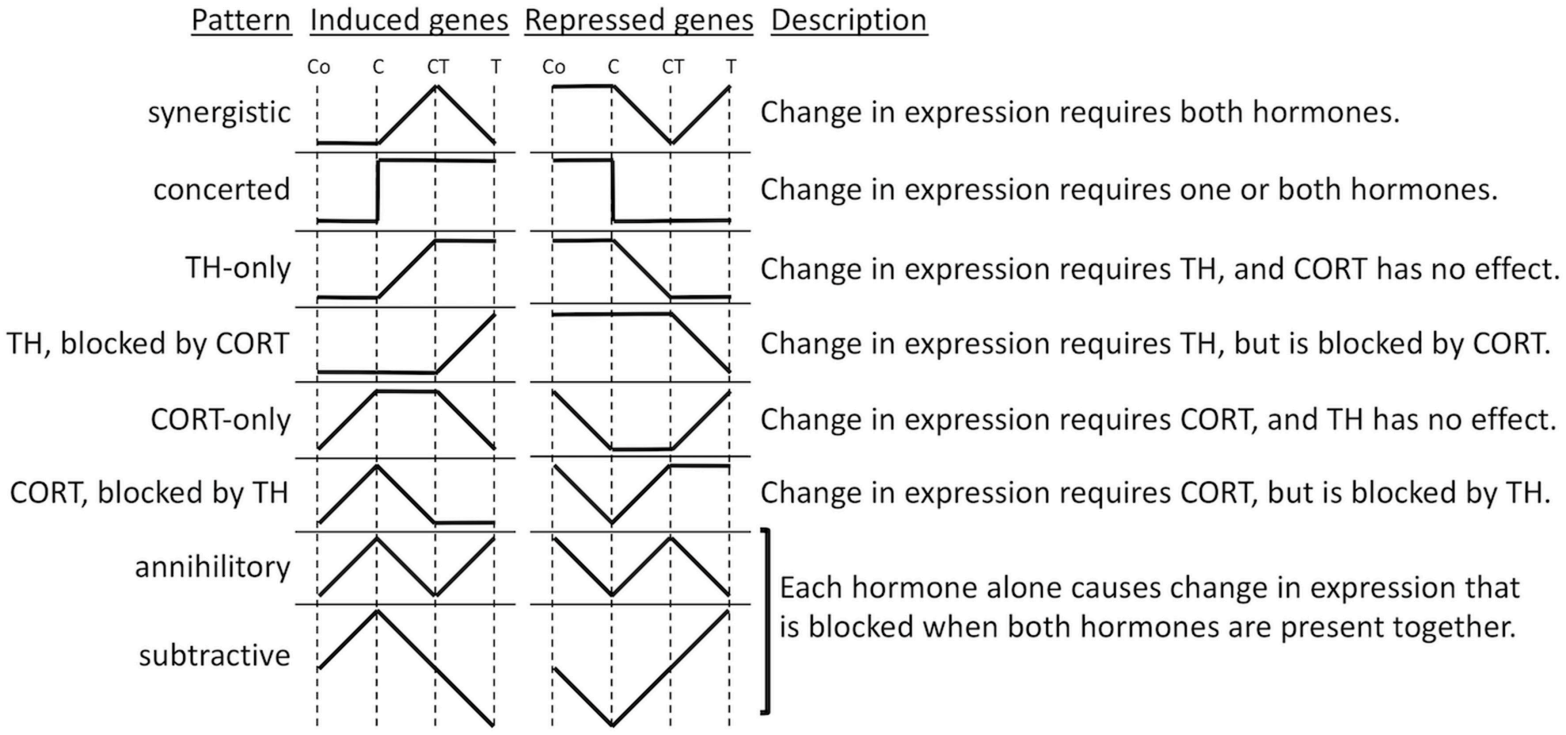
Patterns of gene regulation induced by TH and CORT. Tadpoles were treated with CORT (C), CORT plus TH (CT), or TH (T), or no hormone control (Co), and after 14 h, tails were subjected to microarray analysis. Shown are idealized patterns of changes in gene expression induced by hormone treatments relative to control levels based k-means clustering of significantly regulated genes among treatments (86).

To gain insight into the molecular mechanisms of synergy between TH and GCs, we performed microarray analysis on tail RNA extract from *Xenopus tropicalis* tadpoles treated for 18 h with corticosterone (CORT), T3, CORT plus T3, or vehicle (86). Previously, only one GC response gene was known in tadpoles, i.e., *klf9*, which was also the only known TH/GC synergistic gene. Microarray analysis identified over 5,000 genes whose expression was significantly modified in response to one or more hormone treatments and offered a new opportunity to dissect the interaction between TH and GCs. Cluster analysis led to the identification of numerous patterns of gene regulation (Figure 2). The greatest number of these genes was regulated by T3 unaffected by CORT (33%) and by CORT unaffected by T3 (12%). Noteworthy were these so-called “CORT-only” genes because they represent GC response genes not affected by TH signaling. Many genes either required both hormones together (22%) or were regulated by each hormone separately as well as together (16%), which may represent truly synergistic (require both hormones for expression) and/or some form of additive interaction, like *klf9* where both hormones can contribute individually. The remaining genes (17%) represent some form of antagonism on gene expression, predominantly TH gene regulation blocked by CORT and CORT gene regulation blocked by TH. These antagonistic hormone interactions at the gene expression level contrast sharply with the solely synergistic action of these hormones at the morphological level, i.e., GCs accelerate TH-induced metamorphic change. The tail transcriptome results obtained by DNA arrays showed that the effect of T3 and CORT co-treatment is not simply the addition of T3-regulatd genes plus GC regulated genes. The unexpectedly complex and uncharacterized mechanisms of gene regulation for a large number of genes controlled by TH and GCs represents an open frontier in need of future research to understand how developing organisms interact with the environment to modulate development via altered hormonal input.

### TH and GC Crosstalk: Regulated Gene Functions

To expand the understanding of the hormonal cross talk and link clustering with biological functions, gene ontology (GO) analysis was applied to the gene lists (86). The genes significantly regulated by T3 (Figure 2, TH-only genes) included GO categories, corresponding to programmed cell death and metallopeptidase activity. It makes sense that an increase in T3 would increase the expression of genes involved in tissue resorption. Genes up-regulated by CORT (Figure 2, CORT-only genes) are associated with energy production in mitochondria and metabolic processes. Again, it makes sense that an increase in GCs increases the expression of genes involved in gluconeogenesis to regulate energy requirements for altered metabolism during stress and provide sufficient energy for the acceleration of metamorphosis. Genes synergistically up-regulated when CORT and T3 were present together (Figure 2, synergistic induced genes) include GO terms associated with intracellular protein transport, vesicle-mediated transport, protein localization, and cellular localization. Finally, genes that are down-regulated by T3 and CORT co-treatment (Figure 2, synergistic repressed genes) are linked with negative regulation of development and cell differentiation. Globally, these results are consistent with the action of the two hormones to promote tail resorption. It is important to note, however, that the proportion of genes that emerge from GO analysis is small relative to the number of differentially expressed genes. Thus, the ontology analysis results do not reflect all the functions represented by the differentially expressed genes. There remains, therefore, an important part of the data for which we are not yet in a position to define the biological function.

### TH and GC Crosstalk: Mechanism of Gene Regulation

The number of patterns of TH- and GC-response gene regulation (Figure 2) suggests that multiple molecular mechanisms likely exist to provide this gene regulation diversity. Research into these mechanisms of interest benefits from knowledge that T3 and GCs act directly through nuclear receptors that initiate gene regulation cascades of induced transcription factors (20, 121). Identifying direct response genes for each hormone is a key element of on-going research. TH and GCs regulate gene expression via hormone response elements (HRE) that interact with the promoter of hormone direct target genes (23, 122). The identification of such HREs is difficult because of the complexity of these elements (123). First, HRE sequences may be partially degenerate, engendering numerous false positives identified by sequence analysis algorithms. Second, the presence of an HRE sequence does not guarantee the binding of the receptor, presumably because the chromatin organization around an HRE can dictate the accessibility of the HRE to receptors. Finally, the HRE position relative to the promoter of the target gene can be near or far upstream or downstream of the gene and also within the gene (124). Despite advances in knowledge and computer algorithms, currently only experimentation can allow the identification of HREs (123).

To date, only 12 direct T3-response genes have an identified HRE in *Xenopus tropicalis* (125), including *thr*β, *klf9, thibz* (a transcription factor), the metalloproteinases *mmp11* and *mmp13*, and *dio3*. Such information is still not available for GC-response genes, except for *klf9*. Note that all known HREs are positive, resulting in up-regulation of the gene in response to the hormone. HREs can be either positive or negative, but the existence of negative HREs remains to be established. Likewise, the existence of an “antagonism module” which would harbor HREs for each hormone but with opposite effect on transcription to explain antagonistic gene regulation interactions between TH and GCs, is not known. However, antagonistic interactions may be indirect due to induced transcription factors or chromatin modifiers affecting hormone response gene expression. Indeed, *sox3, dot1L*, and *de novo DNA methyltransferase 3* are direct T3 response genes that themselves affect chromatin structure and gene expression (126–128).

## Conclusions

Numerous explorations into the hormonal control of frog metamorphosis have revealed the powerful effects of TH on nearly every tissue in the tadpole body. These studies have also identified limitations in our ability to replicate these developmental events using exogenous TH. These limitations may be artifacts of the experimental hormone treatments, or TH may indeed be insufficient to accomplish all of the developmental changes of metamorphosis. The relative ease of eliminating TH to study its role in development is contrasted with the difficulty of selectively removing other hormones, as yet unachieved for GCs, aldosterone, prolactin, that may also play a role in natural development. The advent of gene disruption technologies to produce loss of function mutations in pituitary hormones, steroid synthesizing enzymes, and hormone receptors opens the door for continued advances to understand the roles of other hormones besides TH involved in the complex endocrine mechanisms that control post-embryonic development in amphibians.

## Author Contributions

LS and DB have conceived the presented idea and contributed to the writing of the paper.

### Conflict of Interest Statement

The authors declare that the research was conducted in the absence of any commercial or financial relationships that could be construed as a potential conflict of interest.

## References

[1] Fisher DA Chapter 6: Fetal-perinatal thyroid physiology. In: Eugster EA Pescovitz OH editors. Contemporary Endocrinology: Developmental Endocrinology: From Research to Clinical Practice. Totowa NJ: Humana Press (2002). p. 135–49. 10.1007/978-1-59259-156-5_6

[2] Hadj-Sahraoui N Seugnet I Ghorbel MT Demeneix B Hypothyroidism prolongs mitotic activity in the post-natal mouse brain. Neuroscience Lett. (2000) 280:79–82. 10.1016/S0304-3940(00)00768-0 10686382

[3] Henning SJ Chapter 9: Functional development of the gastrointestinal tract. In: Johnson LR editor. Physiology of the Gastrointestinal Tract, Vol. 9. New York, NY: Raven Press (1987). p. 285–300.

[4] Leloup J Buscaglia M La triiodothyronine, hormone de la metamorphose des Amphibiens. C R Acad Sci Paris Ser D. (1977) 284:2261–3.

[5] McNabb FMA The Hypothalamic-Pituitary-Thyroid (HPT) axis in birds and its role in bird development and reproduction. Crit Rev Toxicol. (2007) 37:163–93. 10.1080/10408440601123552 17364708

[6] Norris DO Vertebrate Endocrinology. New York, NY: Academic Press (2007).

[7] Denver RJ Neuroendocrinology of amphibian metamorphosis. Curr Top Dev Biol. (2013) 103:195–227. 10.1016/B978-0-12-385979-2.00007-1 23347520

[8] Fowden AL Endocrine regulation of fetal growth. Reprod Fertil Dev. (1995) 7:351–63. 10.1071/RD9950351 8606944

[9] Fowden AL Forhead AJ Endocrine interactions in the control of fetal growth. In: Bhatia J Bhutta ZA Kalhan SC editors. Maternal and Child Nutrition: The First 1,000 Days. Basel: Karger (2013). p. 91–102. 10.1159/000348417 23887107

[10] Kaltenbach J Endocrinology of amphibian metamorphosis. In: Gilbert LI Tata JR Atkinson BG editors. Metamorphosis: Postembryonic Reprogramming of Gene Expression in Amphibian and Insect Cells. San Diego CA: Academic Press (1996) p. 403–31. 10.1016/B978-012283245-1/50013-0

[11] White BA Nicoll CS Hormonal control of amphibian metamorphosis. In: Gilbert LI Frieden E editors. Metamorphosis: A Problem in Developmental Biology. New York, NY: Plenum Press (1981). p. 363–96. 10.1007/978-1-4613-3246-6_11

[12] Liggins GC The role of cortisol in preparing the fetus for birth. Reprod Fertil Dev. (1994) 6:141–50. 10.1071/RD9940141 7991781

[13] Dodd MHI Dodd JM The biology of metamorphosis. In: Lofts B editor. Physiology of the Amphibia. New York, NY: Academic Press (1976) p. 467–599. 10.1016/B978-0-12-455403-0.50015-3

[14] Bonett RM Hoopfer ED Denver RJ Molecular mechanisms of corticosteroid synergy with thyroid hormone during tadpole metamorphosis. Gen Comp Endocrinol. (2010) 168:209–19. 10.1016/j.ygcen.2010.03.014 20338173 PMC2912948

[15] Forhead AJ Fowden AL Thyroid hormones in fetal growth and prepartum maturation. J Endocrinol. (2014) 221:R87–R103. 10.1530/JOE-14-0025 24648121

[16] Galton VA Mechanisms underlying the acceleration of thyroid hormone-induced tadpole metamorphosis by corticosterone. Endocrinology. (1990) 127:2997–3002. 10.1210/endo-127-6-2997 2249638

[17] Denver RJ Glennemeier KA Boorse GC Endocrinology of complex life cycles: amphibians. In: Pfaff DW Arnold AP Etgen AM Fahrbach SE Ruben RT editors. Hormones, Brain and Behavior, 2nd ed. San Diego,CA: Academic Press (2009) p. 707–44. 10.1016/B978-008088783-8.00021-8

[18] Kikuyama S Kawamura K Tanaka S Yamamoto K Aspects of amphibian metamorphosis: hormonal control. Int Rev Cytol. (1993) 145:105–48. 10.1016/S0074-7696(08)60426-X 8500980

[19] Kulkarni SS Buchholz DR Corticosteroid signaling in frog metamorphosis. Gen Comp Endocrinol. (2014) 203:225–31. 10.1016/j.ygcen.2014.03.036 24713447

[20] Shi Y Amphibian Metamorphosis: From Morphology to Molecular Biology. New York, NY: Wiley-Liss, Inc (1999).

[21] Buchholz DR Hsia SC Fu L Shi YB A dominant-negative thyroid hormone receptor blocks amphibian metamorphosis by retaining corepressors at target genes. Mol Cell Biol. (2003) 23:6750–8. 10.1128/MCB.23.19.6750-6758.2003 12972595 PMC193935

[22] Buchholz DR Tomita A Fu L Paul BD Shi YB Transgenic analysis reveals that thyroid hormone receptor is sufficient to mediate the thyroid hormone signal in frog metamorphosis. Mol Cell Biol. (2004) 24:9026–37. 10.1128/MCB.24.20.9026-9037.2004 15456876 PMC517898

[23] Das B Matsuda H Fujimoto K Sun G Matsuura K Shi YB Molecular and genetic studies suggest that thyroid hormone receptor is both necessary and sufficient to mediate the developmental effects of thyroid hormone. Gen Comp Endocrinol. (2010) 168:174–80. 10.1016/j.ygcen.2010.01.019 20138179 PMC3426277

[24] Schreiber AM Das B Huang H Marsh-Armstrong N Brown DD Diverse developmental programs of Xenopus laevis metamorphosis are inhibited by a dominant negative thyroid hormone receptor. Proc Natl Acad Sci USA. (2001) 98:10739–44. 10.1073/pnas.191361698 11517345 PMC58545

[25] Buchholz DR Paul BD Fu L Shi YB Molecular and developmental analyses of thyroid hormone receptor function in *Xenopus laevis*, the African clawed frog. Gen Comp Endocrinol. (2006) 145:1–19. 10.1016/j.ygcen.2005.07.009 16266705

[26] Sachs LM Damjanovski S Jones PL Li Q Amano T Ueda S Dual functions of thyroid hormone receptors during Xenopus development. Comp Biochem Physiol B Biochem Mol Biol. (2000) 126:199–211. 10.1016/S0305-0491(00)00198-X 10874167

[27] Shi YB Dual functions of thyroid hormone receptors in vertebrate development: the roles of histone-modifying cofactor complexes. Thyroid. (2009) 19:987–99. 10.1089/thy.2009.0041 19678741 PMC2833175

[28] Allen BM The endocrine control of amphibian metamorphosis. Biol Rev. (1938) 13:1–19. 10.1111/j.1469-185X.1938.tb00505.x

[29] Brown DD Cai L Das B Marsh-Armstrong N Schreiber AM Juste R. Thyroid hormone controls multiple independent programs required for limb development in *Xenopus laevis* metamorphosis. Proc Natl Acad Sci USA. (2005) 102:12455–8. 10.1073/pnas.0505989102 16129821 PMC1194953

[30] Marsh-Armstrong N Cai L Brown DD. Thyroid hormone controls the development of connections between the spinal cord and limbs during *Xenopus laevis* metamorphosis. Proc Natl Acad Sci USA. (2004) 101:165–70. 10.1073/pnas.2136755100 14691251 PMC314156

[31] Mukhi S Mao J Brown DD. Remodeling the exocrine pancreas at metamorphosis in *Xenopus laevis*. Proc Natl Acad Sci USA. (2008) 105:8962–7. 10.1073/pnas.0803569105 18574144 PMC2449347

[32] Mukhi S Horb ME Brown DD. Remodeling of insulin producing beta-cells during *Xenopus laevis* metamorphosis. Dev Biol. (2009) 328:384–91. 10.1016/j.ydbio.2009.01.038 19389350 PMC3863375

[33] Mukhi S Brown DD. Transdifferentiation of tadpole pancreatic acinar cells to duct cells mediated by Notch and stromelysin-3. Dev Biol. (2011) 351:311–7. 10.1016/j.ydbio.2010.12.020 21194527 PMC3394455

[34] Schreiber AM Mukhi S Brown DD. Cell-cell interactions during remodeling of the intestine at metamorphosis in *Xenopus laevis*. Dev Biol. (2009) 331:89–98. 10.1016/j.ydbio.2009.04.033 19409886 PMC2712884

[35] Doyle MJ Maclean N. Biochemical changes in developmentally retarded *Xenopus laevis* larvae I. The lens crystallin transition. J Embryol Exp Morph. (1978) 46:215–22. 702033

[36] Allen BM The results of thyroid removal in the larvae of Rana pipiens. J Exp Zool. (1918) 24:499–519. 10.1002/jez.1400240303

[37] Buchholz DR Hayes TB. Variation in thyroid hormone action and tissue content underlies species differences in the timing of metamorphosis in desert frogs. Evol Dev. (2005) 7:458–67. 10.1111/j.1525-142X.2005.05049.x 16174038

[38] Chang L-T Hsu C-Y The relationship between the age and metamorphic progress and the development of the tadpole ovaries. Proc Natl Sci Counc Republ China. (1987) 11B:211–7.

[39] Hoskins ER Hoskins MM Growth and development of amphibia as affected by thyroidectomy. J Exp Zool. (1919) 29:1–69. 10.1002/jez.1400290102

[40] Rot-Nikcevic I Wassersug RJ. Arrested development in *Xenopus laevis* tadpoles: how size constrains metamorphosis. J Exp Biol. (2004) 207:2133–45. 10.1242/jeb.01002 15143146

[41] Frieden E Just JJ Hormonal responses in amphibian metamorphosis. In: Litwack G editor. Biochemical Actions of Hormones. New York, NY: Academic Press (1970) p. 1–52. 10.1016/B978-0-12-452801-7.50006-7

[42] Etkin W The endocrine mechanism of amphibian metamorphosis, an evolutionary achievement. In: Benson GK Phillips JG editors. Hormones and the Environment. Cambridge: University Press (1970) p. 137–55.

[43] Kollros JJ Mechanisms of amphibian metamorphosis: hormones. Amer Zool. (1961) 1:107–14. 10.1093/icb/1.1.107

[44] Hollar AR Choi J Grimm AT Buchholz DR. Higher thyroid hormone receptor expression correlates with short larval periods in spadefoot toads and increases metamorphic rate. Gen Comp Endocrinol. (2011) 173:190–8. 10.1016/j.ygcen.2011.05.013 21651912 PMC3152253

[45] Kulkarni SS Denver RJ Gomez-Mestre I Buchholz DR. Genetic accommodation via modified endocrine signalling explains phenotypic divergence among spadefoot toad species. Nat Commun. (2017) 8:993. 10.1038/s41467-017-00996-5 29051478 PMC5648835

[46] Gomez-Mestre I Kulkarni S Buchholz DR. Mechanisms and consequences of developmental acceleration in tadpoles responding to pond drying. PLoS ONE. (2013) 8:e84266. 10.1371/journal.pone.0084266 24358352 PMC3865288

[47] Choi J Suzuki KT Sakuma T Shewade L Yamamoto T Buchholz DR Unliganded thyroid hormone receptor alpha regulates developmental timing via gene repression as revealed by gene disruption in *Xenopus tropicalis*. Endocrinol. (2015) 156:735–44. 10.1210/en.2014-1554 PMC4298327 25456067

[48] Choi J Atsuko Ishizuya-Oka A Buchholz DR. Growth, development, and intestinal remodeling occurs in the absence of thyroid hormone receptor alpha in tadpoles of *Xenopus tropicalis*. Endocrinol. (2017) 158:1623–33. 10.1210/en.2016-1955 28323943

[49] Buchholz DR Shi YB. Dual function model revised by thyroid hormone receptor alpha knockout frogs. Gen Comp Endocrinol. (2018) 265:214–8. 10.1016/j.ygcen.2018.04.020 29689262 PMC6087486

[50] Nakajima K Tazawa I Yaoita Y Thyroid hormone receptor α- and α-Knockout xenopus tropicalis tadpoles reveal subtype-specific roles during development. Endocrinol. (2018) 159:733–43. 10.1210/en.2017-00601 29126198

[51] Sakane Y Iida M Hasebe T Fujii S Buchholz DR Ishizuya-Oka A. Functional analysis of thyroid hormone receptor beta in *Xenopus tropicalis* founders using CRISPR-Cas. Biology Open. (2018) 7:bio030338. 10.1242/bio.030338 29358165 PMC5829506

[52] Brown DD Wang Z Kanamori A Eliceiri B Furlow JD Schwartzman R. Amphibian metamorphosis: a complex program of gene expression changes controlled by the thyroid hormone. Recent Prog Horm Res. (1995) 50:309–15. 10.1016/B978-0-12-571150-0.50018-4 7740163

[53] Etkin W The mechanisms of anuran metamorphosis I. Thyroxine concentration and the metamorphic pattern. J Exp Zool. (1935) 71:317–40. 10.1002/jez.1400710208

[54] Etkin W Hypothalamic sensitivity to thyroid feedback in the tadpole. Neuroendocrinol. (1965/66) 1:293–302. 10.1159/000121676

[55] Rose CS Cahill JW. How thyroid hormones and their inhibitors affect cartilage growth and shape in the frog *Xenopus laevis*. J Anat. (2019) 234:89–105. 10.1111/joa.12897 30456781 PMC6284441

[56] Atkinson BG Just JJ. Biochemical and histological changes in the respiratory system of *Rana catesbeiana* larvae during normal and induced metamorphosis. Dev Biol. (1975) 45:151–65. 10.1016/0012-1606(75)90248-1 1081056

[57] Veldhoen N Stevenson MR Helbing CC. Comparison of thyroid hormone-dependent gene responses *in vivo* and in organ culture of the American bullfrog (Rana (Lithobates) catesbeiana) lung. Comp Biochem Phys Part D Genomics and Proteomics. (2015) 16:99–105. 10.1016/j.cbd.2015.09.001 26462067

[58] Shewade LH Schneider KA Brown AC Buchholz DR. *In-vivo* regulation of Krüppel-like factor 9 by corticosteroids and their receptors across tissues in tadpoles of *Xenopus tropicalis*. Gen Comp Endocrinol. (2017) 248:79–86. 10.1016/j.ygcen.2017.02.007 28232027

[59] Etkin W Hormonal control of amphibian metamorphosis. In: Etkin W Gilbert LI editors. Metamorphosis: A Problem in Developmental Biology. New York, NY: Appleton-Century-Croft (1968). p. 313–48.

[60] Kikuyama S Miyakawa M Arai Y Influence of thyroid hormone on the development of preoptic-hypothalamic monoaminergic neurons in tadpoles of *Bufo bufo* japonicus. Cell Tissue Res. (1979) 198:27–33. 10.1007/BF00234831 113102

[61] Miyakawa M Arai Y Kikuyama S. Corticosterone stimulates the development of preoptic catecholamine neurons in tadpoles *Bufo bufo* japonicus. Anat Embryol. (1984) 170:113–5. 10.1007/BF00318994 6440455

[62] Broyles RH Changes in the blood during amphibian metamorphosis. In: Gilbert LI Frieden E editors. Metamorphosis, a Problem in Developmental Biology. New York, NY: Plenum Press (1981). p. 461–90. 10.1007/978-1-4613-3246-6_14

[63] Cohen PP Brucker RF Morris SM Cellular and molecular aspects of thyroid hormone action during amphibian metamorphosis. In: Li CH editor. Hormonal Proteins and Peptides. New York, NY: Academic Press (1978). p. 273–381.

[64] Just JJ Atkinson BG. Hemoglobin transitions in the bullfrog, *Rana catesbeiana*, during spontaneous and induced metamorphosis. J Exp Zool. (1972) 182:271–80. 10.1002/jez.1401820210 4538716

[65] Moss B Ingram VM. Hemoglobin synthesis during amphibian metamorphosis, I. I synthesis of adult hemoglobin following thyroxine administration. J Mol Biol. (1968) 32:493–504. 10.1016/0022-2836(68)90337-9 5644918

[66] Nishikawa A Hayashi H. T3-hydrocortisone synergism on adult-type erythroblast proliferation and T3-mediated apoptosis of larval-type erythroblasts during erythropoietic conversion in *Xenopus laevis*. Histochem Cell Biol. (1999) 111:325–34. 10.1007/s004180050364 10219633

[67] Maclean N Turner S. Adult haemoglobin in developmentally retarded tadpoles of *Xenopus laevis*. J Embryol Exp Morph. (1976) 35:261–6. 939939

[68] Maclean N Jurd RD Electrophoretic analysis of the haemoglobins of *Ambystoma mexicanum*. Comp Biochem Physiol. (1971) 40B:751–5. 10.1016/0305-0491(71)90150-7 5133344

[69] Satoh SJ Wakahara M Hemoglobin transition from larval to adult types in a Salamander (*Hynobius retardatus*) depends on activity of the pituitary gland, but not that of the thyroid gland. J Exp Zool. (1997) 278:87–92. 10.1002/(SICI)1097-010X(19970601)278:2&lt;87::AID-JEZ3&gt;3.0.CO;2-0

[70] Ishizuya-Oka A Shi YB. Molecular mechanisms for thyroid hormone-induced remodeling in the amphibian digestive tract: a model for studying organ regeneration. Dev Growth Differ. (2005) 47:601–7. 10.1111/j.1440-169X.2005.00833.x 16316405

[71] Dauça M Hourdry J Hugon JS Ménard D Amphibian intestinal brush border enzymes during thyroxine-induced metamorphosis: a biochemical and cytochemical study. Histochem. (1980) 70:33–42. 10.1007/BF00508844 6970191

[72] Ishizuya-Oka A Shimozawa A. Induction of metamorphosis by thyroid hormone in anuran small intestine cultured organotypically *in vitro*. In Vitro Cell Dev Biol. (1991) 27A:853–57. 10.1007/BF02630987 1748625

[73] Pouyet JC Hourdry J Effet de la thyroxine sur la prolifération des épithéliocytes intestinaux en culture organotypique, chez la larve du crapaud-accoucheur (*Alytes obstetricans* Laurenti). Biol Cell. (1980) 38:237–42.

[74] Pouyet JC Hourdry J. *In vitro* study of the intestinal brush border enzyme activities in developing anuran amphibian: effects of thyroxine, cortisol, and insulin. J Exp Zool. (1988) 245:200–5. 10.1002/jez.1402450209 3130458

[75] Ben Brahim O Mesnard J Hourdry J Hormonal control of the intestinal brush border enzyme activities in developing anuran amphibians. II. Effects of glucocorticoids and insulin during experimental metamorphosis. GCE. (1987) 65:489–95. 10.1016/0016-6480(87)90135-3 3104133

[76] El Maraghi-Ater H Mesnard J Hourdry J Hormonal control of the intestinal brush border enzyme activities in developing anuran amphibians: I. Effects of hydrocortisone and insulin during and after spontaneous metamorphosis. Gen Comp Endocrinol. (1986) 61:53–63. 10.1016/0016-6480(86)90248-0 2867004

[77] Milano EG Chimenti C. Morphogenesis of the pancreas of *Bufo bufo* during metamorphosis. Gen Comp Endocrinol. (1995) 97:239–49. 10.1006/gcen.1995.1023 7622018

[78] Frye BE. Metamorphic changes in the blood sugar and the pancreatic islets of the frog, *Rana clamitans*. J Exp Zool. (1964) 155:215–24. 10.1002/jez.1401550208 14131456

[79] Atkinson BG Little GH Growth and regression in tadpole pancreas during spontaneous and thyroid hormone-induced metamorphosis. Mech Aging Develop. (1972) 1:299–312. 10.1016/0047-6374(72)90075-9

[80] Kim K Slickers KA Biochemistry of anuran pancreas development during thyroxine-induced metamorphosis. In: Hamburgh M Barrington EJW editors. Hormones in Development. New York, NY: Appleton Century Crofts (1971). p. 321–34.

[81] Frye BE. Hypertrophy of the islets of langerhans of frog tadpoles after partial pancreatectomy. J Exp Zool. (1965) 158:133–40. 10.1002/jez.1401580202 14327183

[82] Derby A. An *in vitro* quantitative analysis of the response of tadpole tissue to thyroxine. J Exp Zool. (1968) 168:147–56. 10.1002/jez.1401680203 5692695

[83] Kikuyama S Niki K Mayumi M Shibayama R Nishikawa M Shintake N. Studies on corticoid action on the toad tadpole tail *in vitro*. Gen Comp Endocrinol. (1983) 52:395–9. 10.1016/0016-6480(83)90178-8 6421652

[84] Kikuyama S Niki K Mayumi M Kawamura K. Retardation of thyroxine-induced metamorphosis by Amphenone B in toad tadpoles. Endocrinol Jpn. (1982) 29:659–62. 10.1507/endocrj1954.29.659 6221924

[85] Nishikawa A Kaiho M Yoshizato K. Cell death in the anuran tadpole tail: thyroid hormone induces keratinization and tail-specific growth inhibition of epidermal cells. Dev Biol. (1989) 131:337–44. 10.1016/S0012-1606(89)80007-7 2463945

[86] Kulkarni SS Buchholz DR. Beyond synergy: corticosterone and thyroid hormone have numerous interaction effects on gene regulation in *Xenopus tropicalis* tadpoles. Endocrinol. (2012) 153:5309–24. 10.1210/en.2012-1432 22968645

[87] Little GH Castro CE Taurine levels in the anuran tadpole tail during spontaneous and triiodothyronine-induced metamorphosis. Comp Biochem Physiol. (1976) 54A:245–7. 10.1016/S0300-9629(76)80105-3 4276

[88] Kubler H Frieden E. The increase in beta-glucouronidase of the tadpole tail during anuran metamorphosis and its relation to lysosomes. Biochim Biophys Acta. (1964) 93:635–43. 10.1016/0304-4165(64)90346-0 14263161

[89] Wang VB Frieden E. Changes in cathepsin C activity during spontaneous and induced metamorphoses of the bullfrog. Gen Comp Endocrinol. (1973) 21:381–9. 10.1016/0016-6480(73)90071-3 4543146

[90] Suzuki K Sato K Katsu K Hayashita H Bach Kristensen D Yoshizato K. (2001). Novel Rana keratin genes and their expression during larval to adult epidermal conversion in bullfrog tadpoles. Differentiation. 68:44–54. 10.1046/j.1432-0436.2001.068001044.x 11683492

[91] Suzuki K-T Suzuki M Shigeta M Fortriede JD Mawaribuchi S Yamamoto T. Clustered Xenopus keratin genes: a genomic, transcriptomic, and proteomic analysis. Dev. Biol. (2017) 426:384–92. 10.1016/j.ydbio.2016.10.018 27842699

[92] Yoshizato K. Molecular mechanism and evolutional significance of epithelial–mesenchymal interactions in the body- and tail-dependent metamorphic transformation of anuran larval skin. Int Rev Cytol. (2007) 260:213–60. 10.1016/S0074-7696(06)60005-3 17482907

[93] Fox H Amphibian Morphogenesis. Clifton, NJ: Humana Press (1983).

[94] Mukhi S Cai L Brown DD. Gene switching at *Xenopus laevis* metamorphosis. Dev Biol. (2010) 338:117–26. 10.1016/j.ydbio.2009.10.041 19896938

[95] Shimizu-Nishikawa K Miller L. Hormonal regulation of adult type keratin gene expression in larval epidermal cells of the frog *Xenupus laevis*. Differentiation. (1992) 49:77–83. 10.1111/j.1432-0436.1992.tb00771.x 1375919

[96] Utoh R Shigenaga S Watanabe Y Yoshizato K. Platelet-derived growth factor signaling as a cue of the epithelial–mesenchymal interaction required for anuran skin metamorphosis. Dev Dyn. (2003) 227:157–69. 10.1002/dvdy.10302 12761844

[97] Lynn WG Wachowski HE The thyroid gland and its function in cold-blooded vertebrates. Q Rev Biol. (1951) 26:123–68. 10.1086/398076 14854147

[98] Krain LP Denver RJ. Developmental expression and hormonal regulation of glucocorticoid and thyroid hormone receptors during metamorphosis in *Xenopus laevis*. J Endocrinol. (2004) 181:91–104. 10.1677/joe.0.1810091 15072570

[99] Kühn ER De Groef B Grommen SV Van der Geyten S Darras VM. Low submetamorphic doses of dexamethasone and thyroxine induce complete metamorphosis in the axolotl (*Ambystoma mexicanum*) when injected together. Gen Comp Endocrinol. (2004) 137:141–7. 10.1016/j.ygcen.2004.03.005 15158126

[100] Leloup-hatey J Buscaglia M Jolivet-Jaudet G Leloup J Interrenal function during the metamorphosis in anuran amphibia. Fortschr Der Zool. (1990) 38:139–54.

[101] Lorenz C Opitz R Lutz I Kloas W. Corticosteroids disrupt amphibian metamorphosis by complex modes of action including increased prolactin expression. Comp Biochem Physiol Part C. (2009) 150:314–21. 10.1016/j.cbpc.2009.05.013 19481173

[102] Hayes TB. Interdependence of corticosterone and thyroid hormones in larval toads (Bufo boreas). I Thyroid hormone-dependent and independent effects of corticosterone on growth and development. J Exp Zool. (1995) 271:95–102. 10.1002/jez.1402710204 7884391

[103] Kobayashi H Effects of desoxycorticosterone acetate on metamorphosis induced by thyroxine in anuran tadpoles. Endocrinol. (1958) 62:371–7. 10.1210/endo-62-4-371 13524153

[104] Remy C Bounhiol JJ. Normalized metamorphosis achieved by adrenocorticotropic hormone in hypophysectomized and thyroxined Alytes tadpoles. C R Acad Sci Hebd Seances Acad Sci D. (1971) 272:455–8. 4324328

[105] Gereben B Zavacki AM Ribich S Kim BW Huang SA Simonides WS. Cellular and molecular basis of deiodinase-regulated thyroid hormone signaling. Endocr Rev. (2008) 29:898–938. 10.1210/er.2008-0019 18815314 PMC2647704

[106] Hayes TB Wu TH. Interdependence of corticosterone and thyroid hormones in toad larvae (Bufo boreas). II Regulation of corticosterone and thyroid hormones. J Exp Zool. (1995) 271:103–11. 10.1002/jez.1402710205 7884384

[107] Darras VM Van der Geyten S Cox C Segers IB De Groef B Kühn ER. Effects of dexamethasone treatment on iodothyronine deiodinase activities and on metamorphosis-related morphological changes in the axolotl (*Ambystoma mexicanum*). Gen Comp Endocrinol. (2002) 127:157–64. 10.1016/S0016-6480(02)00038-2 12383443

[108] Shi YB Wong J Puzianowska-Kuznicka M Stolow MA. Tadpole competence and tissue-specific temporal regulation of amphibian metamorphosis: roles of thyroid hormone and its receptors. Bioessays. (1996) 18:391–9. 10.1002/bies.950180509 8639162

[109] Choi J Moskalik CL Ng A Matter SF Buchholz DR. Regulation of thyroid hormone-induced development *in vivo* by thyroid hormone transporters and cytosolic binding proteins. Gen Comp Endocrinol. (2015) 222:69–80. 10.1016/j.ygcen.2015.07.006 26188717

[110] Nakajima K Fujimoto K Yaoita Y. Regulation of thyroid hormone sensitivity by differential expression of the thyroid hormone receptor during Xenopus metamorphosis. Genes to Cells. (2012) 17:645–59. 10.1111/j.1365-2443.2012.01614.x 22686326

[111] Tata JR Baker BS Machuca I Rabelo EM Yamauchi K. Autoinduction of nuclear receptor genes and its significance. J Steroid Biochem Mol Biol. (1993) 46:105–19. 10.1016/0960-0760(93)90286-6 8664159

[112] Bagamasbad PD Bonett RM Sachs L Buisine N Raj S Knoedler JR. Dicephering the regulatory logic of an ancient, ultraconserved nuclear receptor enhancer module. Mol Endocrinol. (2015) 29:856–72. 10.1210/me.2014-1349 25866873 PMC4447637

[113] Suzuki MR Kikuyama S. Corticoids augment nuclear binding capacity for triiodothyronine in bullfrog tadpole tail fins. Gen Comp Endocrinol. (1983) 52:272–8. 10.1016/0016-6480(83)90122-3 6197340

[114] Schneider MJ Galton VA. Effect of glucocorticoids on thyroid hormone action in cultured red blood cells from *Rana catesbeiana* tadpoles. Endocrinology. (1995) 136:1435–40. 10.1210/endo.136.4.7895654 7895654

[115] Hoopfer ED Huang L Denver RJ. Basic transcription element binding protein is a thyroid hormone-regulated transcription factor expressed during metamorphosis in *Xenopus laevis*. Dev Growth Differ. (2002) 44:365–81. 10.1046/j.1440-169X.2002.00650.x 12392570

[116] Knoedler JR Denver RJ. Krüppel-like factors are effectors of nuclear receptor signaling. Gen Comp Endocrinol. (2014) 203:49–59. 10.1016/j.ygcen.2014.03.003 24642391 PMC4339045

[117] Bonett RM Hu F Bagamasbad P Denver RJ. Stressor and glucocorticoid-dependent induction of the immediate early gene kruppel-like factor 9: implications for neural development and plasticity. Endocrinology. (2009) 150:1757–65. 10.1210/en.2008-1441 19036875 PMC2659263

[118] Denver RJ Williamson KE. Identification of a thyroid hormone response element in the mouse Krüppel-like factor 9 gene to explain its postnatal expression in the brain. Endocrinology. (2009) 150:3935–43. 10.1210/en.2009-0050 19359381 PMC2717889

[119] Bagamasbad P Howdeshell KL Sachs LM Demeneix BA Denver RJ. A role for basic transcription element-binding protein 1 (BTEB1) in the autoinduction of thyroid hormone receptor beta. J Biol Chem. (2008) 283:2275–85. 10.1074/jbc.M709306200 18045867

[120] Hu F Knoedler JR Denver RJ. A mechanism to enhance cellular responsivity to hormone action: Krüppel-like factor 9 promotes thyroid hormone receptor-β autoinduction during postembryonic brain development. Endocrinol. (2016) 157:1683–93. 10.1210/en.2015-1980 26886257 PMC4816725

[121] Grimaldi A Buisine N Miller T Shi Y-B Sachs LM. Mechanisms of thyroid hormone receptor action during development: lessons from amphibian studies. Biochim Biophys Acta. (2013) 1830:3882–92. 10.1016/j.bbagen.2012.04.020 22565053

[122] Wong J Shi YB. Coordinated regulation of and transcriptional activation by Xenopus thyroid hormone and retinoid X receptors. J Biol Chem. (1995) 270:18479–83. 10.1074/jbc.270.31.18479 7629175

[123] Grimaldi AG Buisine N Bilesimo P Sachs LM. High-throughput sequencing will metamorphose the analysis of thyroid hormone receptor function during amphibian development. Curr Topics Dev Biol. (2013) 103:277–303. 10.1016/B978-0-12-385979-2.00010-1 23347523

[124] Buisine N Ruan X Bilesimo P Grimaldi A Alfama G Ariyaratne P. *Xenopus tropicalis* geneome re-scaffolding and re-annotation reach the resolution required for *in vivo* ChIA-PET analysis. PLoS ONE. (2015) 10:e0137526. 10.1371/journal.pone.0137526 26348928 PMC4562602

[125] Das B Heimeier RA Buchholz DR Shi YB. Identification of direct thyroid hormone response genes reveals the earliest gene regulation programs during frog metamorphosis. J Biol Chem. (2009) 284:34167–78. 10.1074/jbc.M109.066084 19801647 PMC2797187

[126] Kyono Y Sachs LM Bilesimo P Wen L Denver RJ. Developmental and thyroid hormone regulation of the DNA methyltransferase 3a gene in Xenopus tadpoles. Endocrinology. (2016) 157:4961–72. 10.1210/en.2016-1465 27779916 PMC5133355

[127] Matsuura K Fujimoto K Fu L Shi YB. Liganded thyroid hormone receptor induces nucleosome removal and histone modifications to activate transcription during larval intestinal cell death and adult stem cell development. Endocrinol. (2012) 153:961–72. 10.1210/en.2011-1736 22147009 PMC3275393

[128] Sun G Fu L Wen L Shi YB. Activation of Sox3 gene by thyroid hormone in the developing adult intestinal stem cell during Xenopus metamorphosis. Endocrinol. (2014) 155:5024–32. 10.1210/en.2014-1316 25211587 PMC4239430

